# Progress in Medicine

**Published:** 1898-02-26

**Authors:** 


### Progress in Medicine.

RENAL MEDICINE (*concluded*).

**Renal Tuberculosis.**—Kelynack[9] gives some statistics from the post-mortem room at Manchester, where he found only 20 cases of scrofulous kidneys in 4,500 post-mortems, 16 being males and 4 females. The kidneys were enlarged in most cases, the average weight being over 7 oz. Pulmonary tubercle also existed in 14 out of the 20 cases. Meningitis, tuberculosis of the intestines, of the vertebræ, and of the liver were also found, each in a single instance. Tubercle of the testis and prostate occurred rather more frequently, and, in short, in 18 out of the 20 cases some manifestation of tubercle was found elsewhere, and both kidneys were involved in the majority of instances. Hence the importance of a thorough examination of the body before surgical intervention. Miliary tuberculosis, as opposed to the scrofulous or caseating disease is still more often secondary to the disease elsewhere, and affects, as a rule, both kidneys. D. D. Stewart,[10] however, reports an instance in a female, aged 45, who was also the subject of chronic nephritis, of primary miliary tuberculosis affecting one kidney only. The urine showed a tenth albumen, with pus and tubercle bacilli; but death took place from the nephritis. Indeed, miliary tuberculosis of the kidney often gives rise to no distinctive symptoms apart from those of the disease elsewhere, until the mucous membranes of the pelvis, the calices, or of the ureters are inflamed. None were observed in the author's case even after the pelvis was affected, and in a second patient still living the only symptoms were some pus in the urine, among which, after a long search and 14 examinations, bacilli were found. The ordinary symptoms of tuberculous disease of the kidney, such as dysuria, blood, lumbar pain with pus in the water, are usually only too well marked, but it is well to remember that any or all of them may be wanting.

**Treatment.**—Nicolaer,[11] who introduced urotropine into therapeutics, writes again in its favour. It has great solvent powers upon uric acid, and destroys micro-organisms in cystitis where ammoniacal fermentation has taken place. The drug is found undecomposed in the urine a quarter of an hour after administration. He recommends not more than 22 grains daily; indeed, in cystitis he reduces the amount after two days to 15 grains in three doses. This quantity is usually well borne for a long while, but large doses, if continued, produce pain and finally hæmaturia. The cystitis is soon modified, the odour abates and the reaction becomes acid, and the urine becomes clear, though some pus may remain for a time. He has observed this effect even where injections with other active treatment failed to cure the cystitis. Lycetol as a diuretic and solvent of uric acid is reported upon by P. Hamonic,[12] who finds that it moderates the severity of renal colic and removes the brickdust precipitate. Still he does not claim that it will dissolve a large stone, especially one of the mixed sort. It is contraindicated in phosphaturia, but in sixteen cases of purulent cystitis it exercised a favourable action, better indeed than was observed with salol, especially as it retards the decomposition of the urine. No injurious effects of the drug have been noticed.

For the relief of renal headache with a high tension pulse Donald Hood[13] recommends the continuous use of small doses of bromide with large ones of carbonate of potash and ammonia, given as an effervescing draught. Gallois[14] also describes a mode of treating that obstinate malady uræmic dyspnœa, which has given good results where a moderate amount of kidney tissue is still available. He employs very large doses of ether, amounting to two or three ounces in the twenty-four hours. Thirty drops are given at a time in deep injections alternately with a dessert spoonful by the mouth. The relief is very marked, and usually diuresis sets in after a few hours. The treatment must be continued for several days, the intervals between the doses being increased as the dyspnœa improves. The drug may be discontinued after a week if the diuresis be regular. Bronovsky[15] reports further investigations on strontium lactate, and finds that it lowers the tension, the respiration is unaltered, and the pulse is at first slowed and then quickened. In some forms of Bright's disease it has a diuretic action, and diminishes the amount of albumen, but it is improbable that it has any direct action on the renal epithelium. With enormous doses in animals renal irritation and hæmaturia are produced. Bovet and Huchard[16] show the value of repeated saline injections in "surgical kidney." Different amounts were injected during a month in the case of a patient who had passed pus and albumen for ten months and finally sustained a chill. When the injections were increased to 1,700 to 2,000 grammes daily, with copious enemata, a rapid improvement and final recovery took place. The injections were subcutaneous, and as much as 62 ounces were injected in one place at once, and absorbed within an hour. Daily repetition of this dose presented no dangerous symptoms. Albumen, urobilin, and pus diminished with the increase of the injections. In another patient with uræmia, anasarca, dyspnœa, and rather scanty urine, similar enormous injections were given with temporary relief, but the urine decreased, and in a few days death followed. They consider that saline injections are contraindicated when affections of the heart exist, and other writers think them dangerous if the kidney is seriously crippled, unless accompanied with venesection. It is clear, however, from this remarkable case, that even such enormous injections may be of the highest value if the kidneys are sufficiently active to respond to them. It is noticeable that only moderate injections were used at first. These showed the sufficiency of the kidneys, and some improvement from the treatment, and not till then were the massive injections employed which brought about a cure so rapidly.

The dependence of renal diseases upon a cool, damp climate is discussed by Danforth,[17] who notices their remarkable prevalence in Holland, Denmark, and Newfoundland, and their comparative absence in New Zealand, the Mediterranean, Mauritius, the Bermudas, and South Africa. Again, while lardaceous disease is the most common form seen in the tropics, it is remarkable that parenchymatous and interstitial nephritis are almost unknown in the arctic regions, and the suggestion is made that this is owing to the increased activity of the respiratory function for the purpose of maintaining the body heat, and the greatly increased consumption of fats in place of nitrogenous foods. The small consumption of animal food in the tropics may be one of the causes of the absence of kidney diseases other than lardaceous; but the writer admits that the British soldier shows much the same immunity in the tropics, though his food is not greatly altered from that which he receives at home. Malarial infection is, he believes, not infrequently the cause of parenchymatous nephritis; but, if so, it apparently disappears under quinine.

[9] Med. Chron., May. [10] Med. News, Aug. 14. [11] Interl. Med. Mag., Oct. [12] N.Y. Med. Jour. May. [13] "Treatment," Aug. 26. [14] B.M.J., Sept. 18. [15] B.M.J., Sept. 4. [16] Amer, Med.-Surg. Bulletin, Aug. [17] Inter. Med. Mag., Oct.

